# Efficient extraction method to collect sugar from sweet sorghum

**DOI:** 10.1186/1754-1611-7-1

**Published:** 2013-01-10

**Authors:** Fei Jia, Jeerwan Chawhuaymak, Mark R Riley, Werner Zimmt, Kimberly L Ogden

**Affiliations:** 1 Department of Agricultural and Biosystems Engineering, The University of Arizona, Tucson, AZ, 85721, USA; 2 Department of Chemical and Environmental Engineering, The University of Arizona, Tucson, AZ, 85721, USA; 3 Current address: Department of Biological Systems Engineering, University of Nebraska-Lincoln, 223 LW Chase Hall, Lincoln, NE, 68583-0726, USA

**Keywords:** Sweet sorghum, Sugar extraction, Biomass storage, Ethanol fermentation

## Abstract

**Background:**

Sweet sorghum is a domesticated grass containing a sugar-rich juice that can be readily utilized for ethanol production. Most of the sugar is stored inside the cells of the stalk tissue and can be difficult to release, a necessary step before conventional fermentation. While this crop holds much promise as an arid land sugar source for biofuel production, a number of challenges must be overcome. One lies in the inherent labile nature of the sugars in the stalks leading to a short usable storage time. Also, collection of sugars from the sweet sorghum stalks is usually accomplished by mechanical squeezing, but generally does not collect all of the available sugars.

**Results:**

In this paper, we present two methods that address these challenges for utilization of sweet sorghum for biofuel production. The first method demonstrates a means to store sweet sorghum stalks in the field under semi-arid conditions. The second provides an efficient water extraction method that can collect as much of the available sugar as feasible. Operating parameters investigated include temperature, stalk size, and solid–liquid ratio that impact both the rate of sugar release and the maximal amount recovered with a goal of low water use. The most desirable conditions include 30°C, 0.6 ratio of solid to liquid (w/w), which collects 90 % of the available sugar. Variations in extraction methods did not alter the efficiency of the eventual ethanol fermentation.

**Conclusions:**

The water extraction method has the potential to be used for sugar extraction from both fresh sweet sorghum stalks and dried ones. When combined with current sugar extraction methods, the overall ethanol production efficiency would increase compared to current field practices.

## Background

With the increasing demand for fuel and the depletion of fossil resources, ethanol production has increased greatly as an alternative transportation fuel. Ethanol is predominantly produced through fermentation of carbohydrates extracted from sugar-rich plants such as corn, sweet sorghum, sugar cane, and others. Corn is the traditional feedstock for ethanol production due to its high starch content and well-developed infrastructure for growth and processing. However corn ethanol production is limiting because corn serves also as a predominant food for animals and humans, it requires sizeable amounts of fertilizer and water, and it does not grow efficiently in some drier climates.

**Figure 1:**
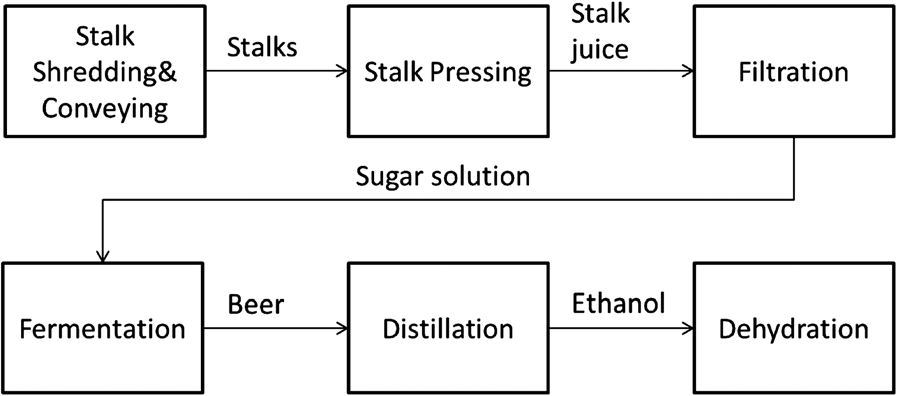
Typical sweet sorghum ethanol fermentation process.

Sweet sorghum (*Sorghum bicolor* (L.) Moench) has been considered as a potential ethanol production feedstock because it accumulates fermentable sugar in the stalk [1] as well as having resistance to drought and tolerance of high salinity soil. Sweet sorghum juice usually contain approximately 16–18% fermentable sugars which are mainly comprised of sucrose, glucose and fructose. Ethanol yield in multiple locations has reportedly ranged from 2129 L ha^−1^ in Michigan to 6388 L ha^−1^ in Hawaii [2]. A typical ethanol production process from sweet sorghum is shown in Figure 1. Ethanol yield and biomass concentration were enhanced by fed-batch fermentation from sweet sorghum juice [3]. Challenges persist for collecting sugar from the stalks. 

The traditional method to extract sugar from sweet sorghum is to squeeze the stalks through a roller mill, releasing the sugar rich juice in a process derived from sugar cane sugar extraction. The main drawbacks of crushing are: 1) there is substantial fermentable sugar remaining after a single crushing (less than half of the total sugar in the stalks typically is recovered) [4][5] and 2) it is labor and energy intensive. Although high sugar extraction efficiency (95%) can be achieved by multi-staged, immobile extraction technologies used to process sugarcane [6], more energy is expended to achieve such extraction efficiency [7]. Juice extraction and sugar recovery increase with reduced roll gap, but tighter crushing leads to more frequent blockage in the mill. Sugar recovery has been shown to increase by shredding the stalks before crushing and by adding water during the squeezing process [8].

We present here a water extraction method similar to that which has been used for sugar extraction from sugar beets, cashew apple bagasse, and carob [8-10]. Extraction of sugar from each agricultural source requires unique operating conditions developed based on sugar and water content, fiber structure and composition, and geometric size. For example, to extract sugar from cashew apple bagasse, the optimum extraction conditions include a volume to mass ratio of liquid: solid of 1: 3.26 (mL/g), pH 6.42, extraction time of 6.3 h and a temperature of 52°C [8]. One drawback of the water extraction method is that the sugar concentration in the extraction water typically is fairly low making it difficult to meet the desired operating condition of 20 Brix (g sucrose/ 100 ml solution) for industrial scale ethanol production. In sugar beet sugar production, press water was completely recycled to the extraction of sugar beet cossettes to increase the sugar concentration [11]. Improved water extraction methods were developed and assessed in this research to overcome the low sugar concentration and high water requirement.

The goal of this research is to develop and characterize a means to collect fermentable sugar from sweet sorghum stalks, which can be used as feedstock for ethanol production. Biological engineering methods incorporating an understanding of plant physiology, engineering mechanics and separations, and fermentations were utilized. The method can collect much of the plant sugars and be utilized either with fresh stalks, with highly desiccated stalks, or with processed biomass.

## Results

### Effect of storage condition

**Figure 2:**
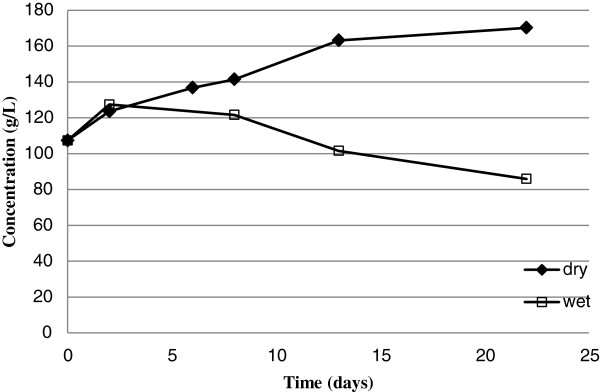
Sugar concentrations of sweet sorghum juice from stalks stored under dry and wet storage conditions.

**Figure 3:**
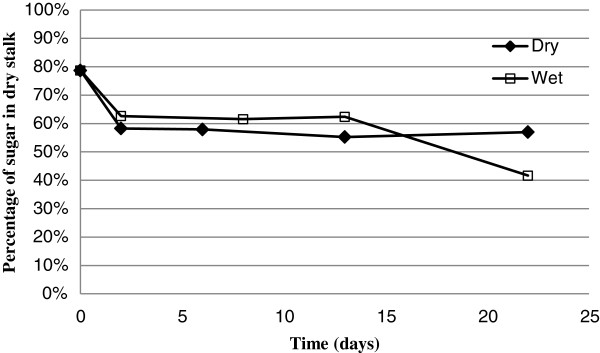
Total sugar mass against dry stalk mass.

Storage of sweet sorghum stalks was performed in two conditions in which water could either evaporate away or with no water loss out of the system (the latter is referred to as a closed condition where any water released from a stalk was retained in contact with the stalks within a plastic bag). Under both dry and wet conditions, there was a 5.0% decrease in water content of the stalks over the first 2 days (data not shown). Between days 2 to 13, the water content of dry storage stalks dropped from 88% to 77%, while the water content of the wet stored stalks did not change appreciably from day 2 to day 22. The sugar concentration in stalks that were stored dry increased from 107 g/L to 170 g/L due primarily to water loss decreasing the volume (Figure 2) but this is somewhat misleading due to the aforementioned loss in water. Therefore, the total sugar content (mass of sugar, M_sugar_) against dry stalk mass (M_dry stalk_) was calculated assuming that the density of the juice approximately equals that of water. This was calculated using:

(1) $$\frac{M_{\mathrm{sugar}}}{M_{\mathrm{dry}\;\mathrm{stalk}}}=\frac{C_{\mathrm{sugar}\;*\;}\mathrm{Moisture}}{1-\mathrm{Moisture}}$$

with Moisture as a fraction (0 < Moisture < 1) v/v. Total sugar decreased 26% and 20% for dry and wet storage conditions, respectively, in the first 2 days (Figure 3). There was no significant total sugar mass change from day 2 to day 22 for dry stored stalks. The total sugar mass in wet stalks decreased 33% from day 13 to day 22; likely this decrease in sugar mass in wet storage was due to microbial consumption, as gauged based on the strong aromatic smell of wet stalks but which was not observed with dry stalks. 

### Sugar release kinetics

**Figure 4:**
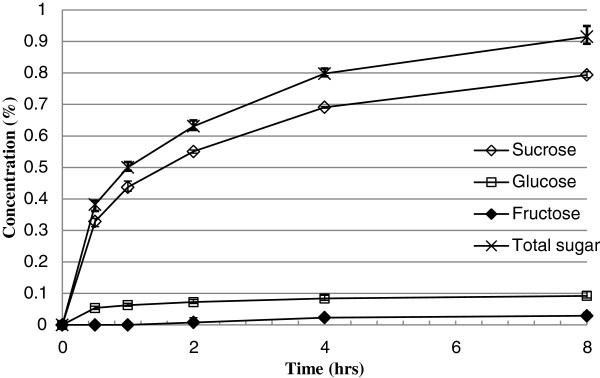
1cm stalk intervals (Size 1) at 25°C sugar extraction (n = 3).

A series of experiments to study the effect of extraction time, temperature and substrate size on stalk sugar removal were performed. Sugars (sucrose, glucose, fructose) were released at rates that were near reciprocal to their concentration (Figure 4). As the surface area-to-volume ratio of substrates increased, the sugar release rates increased. A first order kinetic model adequately fit the relationship between release rate of each sugar and its concentration for each size substrate. 

**Figure 5:**
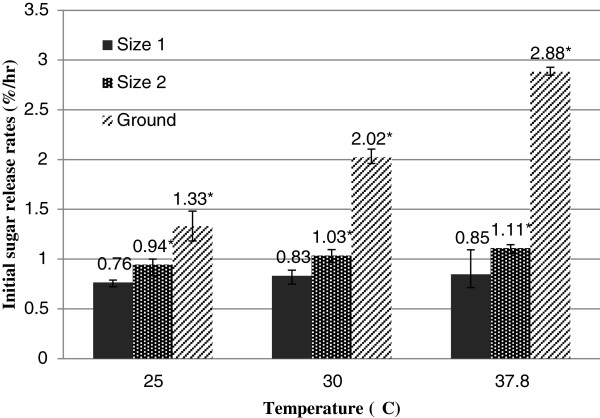
**Effect of substrate size on initial sugar release rate at various temperatures. **Columns labeled with (*) show that there is significant difference in initial sugar release rates between substrate sizes at P < 0.05 for each temperature.

**Figure 6:**
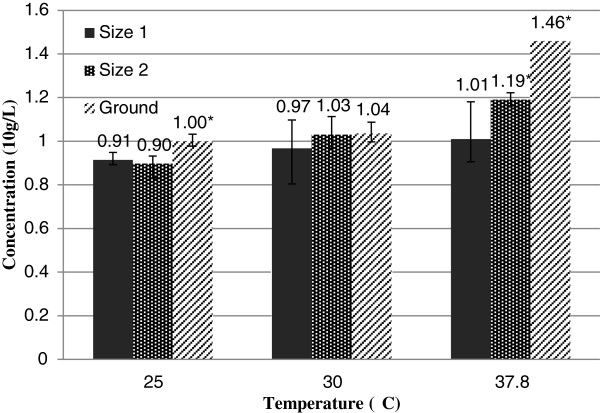
**Effect of substrate size on maximum sugar concentration at various temperatures. **Columns labeled with (*) show that there is significant difference in initial sugar release rates between substrate sizes at P < 0.05 for each temperature.

Initial sugar release rate (measured at the 0.5 h time point for each substrate size) increased with temperature and the degree of stalk mechanical breakdown (Figure 5). Only the ground samples displayed an Arrhenius temperature relationship, while the less processed Size 1 and Size 2 samples showed less of an increase in release rate with temperature than based on an Arrhenius relationship. The maximum amount of sugar released was strongly affected by substrate size with the effects most predominant at 37.8°C (Figure 6). Together, these results suggest that the sorghum fiber structure plays a large role in binding sugar and restricting release. 

### Recycled bagasse sugar extraction

**Figure 7:**
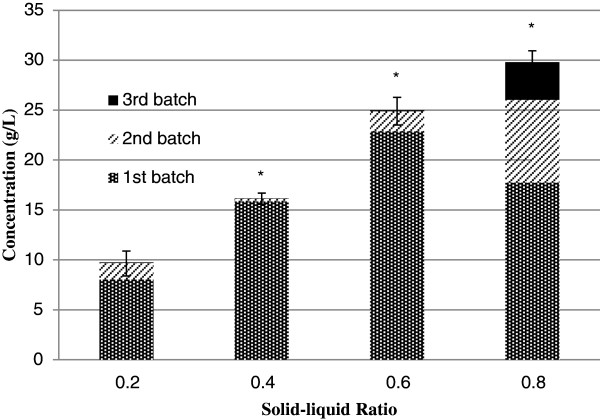
**Total sugar concentrations for each batch of extraction with recycled bagasse. **Columns labeled with (*) show that there is significant difference in total sugar concentration compared to that of 0.2 solid–liquid ratio at P < 0.05.

To collect the maximum amount of sugar from each stalk, repeated extractions using the same sample of bagasse were conducted but with fresh extracting solution (Figure 7). The total amount of sugar released is proportional to the solid–liquid ratio. For solid–liquid ratios between 0.2 and 0.6, 90% of the available sugar was released after one cycle of extraction while a second cycle of extraction captured 99% of the available sugar. Larger solid–liquid ratios of 0.8 had a disproportionate 42% drop in sugar extracted in the first cycle. Such high solid–liquid ratios do not permit continual coverage of the sorghum with extraction water that reduces contact time and hence decreases sugar recovery. A solid–liquid ratio of 0.6 is preferable. 

### Sugar extraction with recycled liquid

**Figure 8:**
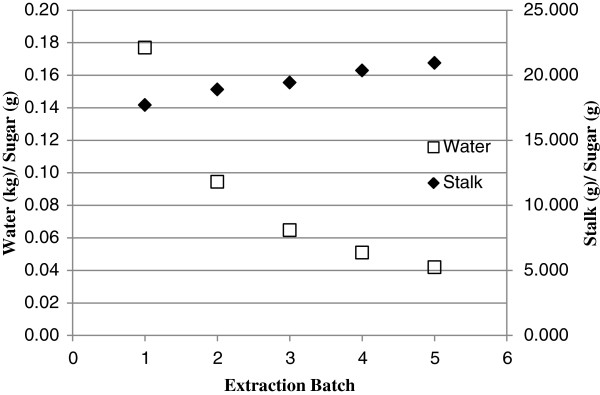
Concentrations of total sugar in recycled water from each batch of extraction.

Water use also must be minimized for this process to be practical. Studies were performed with previously used extraction water with fresh substrate. Thus, sugar extracted from stalks was accumulated and the consumption of water was reduced. The mass of stalks used to obtain a unit mass of sugar increased 18% while the volume of water required decreased 76% at the fifth extraction cycle compared with the first batch and with diminishing returns upon each cycle (Figure 8). Based on stalk and water consumption for a unit of sugar extracted, 5 cycles or less of sugar extraction is recommended. 

### Juice dehydration

Dehydration of sweet sorghum juice has been investigated as a means to reduce shipping costs and to improve stability [12] but over a narrow range of conditions. Fresh juice samples were dehydrated through gentle boiling to reduce the total volume by 10, 20, 30, and 40%. These juice samples were fermented the following day.

**Figure 9:**
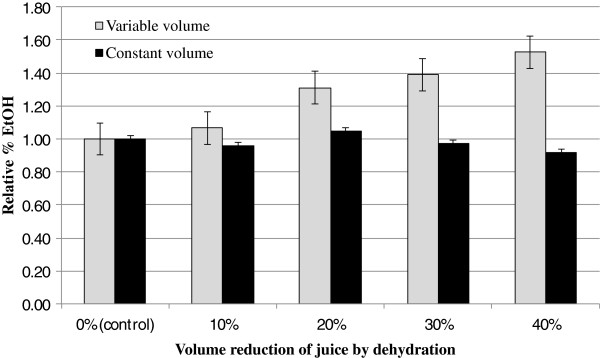
**Relative amount of ethanol concentration comparing with the control group by dehydration of the juice at different concentration. **“Variable volume” samples began with the same starting volume of juice that was reduced by water removal. “Constant volume” measurements are the same experiments, but data normalized to account for the decreased volume fermented. Error bars represent standard error of measurements.

There was no significant difference between 10% dehydrated juice with the control group, but there were significant difference between 20%, 30%, 40% dehydrated juice at P < 0.01, 0.05, and 0.01 (Figure 9), respectively. High yields of ethanol were obtained for the 40% reduction in volume that represents a 50% increase in ethanol concentration (v/v) compared with fully hydrated controls. However, when presented on a mass basis of ethanol (determined using a constant volume assumption) dehydration by 40% *reduces* the ethanol production by 10% compared to unaltered controls. Considering the mass of ethanol produced by comparing percentage of ethanol based on the same initial volume of sweet sorghum juice before dehydration, there is no significant difference (P < 0.05) between treatments of 30% reduction and less and the control. These results suggest that dehydrating the sweet sorghum juice could be a productive method to reduce shipping and storage costs as long as the volume reduction is less than 30%. 

### Juice filtration and fermentation

**Figure 10:**
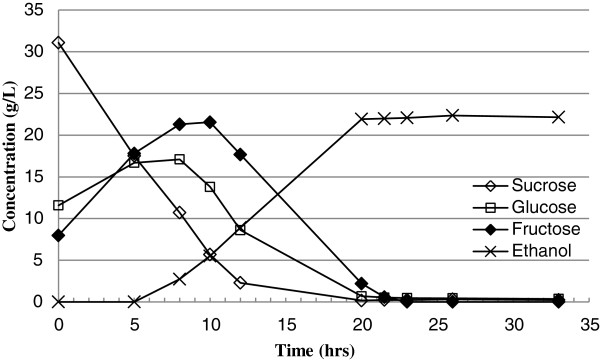
Sugar and ethanol concentration change during extraction solution fermentation.

Juice filtration is a commonly applied field process to clean the juice, but may remove sugars that could be fermented. The ethanol yield and fermentation efficiency from raw juice and filtered juice show no appreciable difference (Table 1). It appears that filtration is not necessary but also does not cause any decrease in ethanol yield. This also was in accordance with the results from Coble [13]. An example fermentation of filtered juice is shown in Figure 10. Note that the rapid decrease of sucrose coincides with an increase in glucose and fructose that are the components of the disaccharide sucrose. As the fermentation proceeded, sucrose was depleted first followed by glucose and fructose being the last sugar depleted. 

**Table 1:** Ethanol yield and fermentation efficiency for juice and extraction water (no replication conducted on these experiments)

**Juice used for fermentation**	**Sugar concentration (g/L)**	**Ethanol yield Y**_**E/S**_**(g ethanol/g sugar)**	**Fermentation efficiency**
Juice without filtration	94.7	0.428	83.8%
Juice filtered with Poly 2000	97.2	0.429	84.1%
Juice filtered with Poly 2004	95.9	0.433	84.9%
Juice filtered with Poly 2007	95.5	0.434	85.2%
Sugar extraction liquid	50.7	0.437	85.8%

Note that the theoretical maximum ethanol production from sugar is a Y_E/S_ of 0.51.

### Feasibility of sugar extraction methods

The efficiency of sugar collection from the standard pressing or crushing method can be compared to that of the water extraction method (Table 2). Taking a typical sweet sorghum stalk, of length 304 cm, and crushing with a commercial field scale press yields juice of 140 mL with a total sugar concentration of 100 g / L. The water extraction method shown here generally works with pieces of stalk of 4 cm length placed in 50 mL of extraction water and yields a sugar concentration of about 9.2 g / L. Comparing these two methods (Table 2) on an amount of sugar collected per amount of stalk used (grams of sugar / stalk length in cm), the water extraction method collects 0.115 g of sugar per cm of stalk while the crushing method yields 0.046 g of sugar per cm of stalk. The water extraction collects approximately 150% greater sugar mass than does the crushing process alone. Certainly the water extraction will require greater energy in water removal, however the recycling of water in the extraction can reduce this amount while reaching nearly the same collection efficiency as the single pass water extraction. 

**Table 2:** Sugar extraction efficiency in crushing and water extraction method with and without recycling of water

	**Stalk length (cm)**	**Juice/water volume (ml)**	**Sugar concentration (g/L)**	**Mass of sugar extracted per unit stalk (Sugar (g)/ stalk (cm))**
Press Juice	304	140	100	0.046
Water extraction with no recycling	4	50	9.2	0.115
Water extraction with recycled water	12	50	25	0.104

## Discussion

Several methods have been explored here for processing sweet sorghum stalks to collect as much sugar as possible and to provide timely utilization of the sugar to generate ethanol using approaches that could integrate well within a full scale ethanol production facility.

Stalk storage in the field was tested with stalks kept enclosed (preventing evaporation of water, leading to what we term a wet condition) and open in which water could evaporate freely. The wet condition was less favorable since the presence of water permitted growth of spontaneous (not purposefully inoculated) microbial breakdown resulting in a sweet, acrid smell of the stalks. In a dry environment, stalks can be stored in the field with about one fourth loss of sugar similar to the results reported by Schmidt [14]; this in field storage strategy could facilitate harvesting logistics. Compared with sugar loss in juice after 3 days storage at room temperature [12], there appears be lower sugar loss in stalks. Within the window between 2 days from harvest until 22 days, there is no significant sugar loss under dry storage in an arid environment.

A key step in the development and testing of the water extraction method is evaluating the relationship between stalk size (amount of mechanical processing) and sugar release kinetics. As anticipated, the time required to reach a maximum sugar concentration decreased as the substrate size decreased. Sugar release rate and total amount of sugar released from sweet sorghum stalks was limited by the size and processing of substrates. Ground stalk allowed sugar to readily diffuse into water at a high rate that followed an Arrhenius relationship with temperature. Stalks that were not ground had substantially less of a temperature dependence that suggests that movement of sugar out of the plant fiber bundles is to some degree inhibited and driven by a process other than diffusion. Certainly, further chopping of the stalks increases the processing energy and cost and together needs to balance with extraction time and cost.

As the sugar concentration in the extraction water increased, the sugar release rate decreased. The unexpected high sugar release rate at the lowest sugar concentration (earliest time) may be caused by the relatively quick sugar diffusion from the surface of the substrates rather than that from internal portions of the substrates. The surface area-to-volume ratio of Size 2 substrate is 72% higher than that of Size 1 substrate; however, the sugar release rate at the lowest sugar concentration in Size 2 substrate increased 14% compared to that in Size 1 substrate. The majority of sugar stored in stalk tissue diffused into water through vascular tissue by the cross section of cut stalk. Sugar inside the stalks appears to have been blocked by stalk tissue in Size 1 and Size 2 substrates. Vascular and fiber tissue were broken up in “Ground” stalk; this eliminated the barrier effect while also increasing surface area.

As liquid reuse cycles increased, less sugar from newly added ground stalks diffused into extraction liquid. This can be explained by the effect of initial sugar concentration on the sugar diffusion rate. The sugar release rate decreased as more sugar accumulated in the extraction liquid. The recycled water extraction method is preferable since it extracts more sugar and uses less than the recycled bagasse extraction method.

The increase of glucose and fructose concentration during early stage of the fermentation was caused by the breakdown of sucrose by sucrase in yeast. There is no discernible effect on ethanol fermentation efficiency from the water extraction process.

Although the sugar concentration in the water extraction method is low compared with that from the crushing method, 2.5 times more sugar mass was recovered from sweet sorghum stalks by the water extraction method. Furthermore, the recycled water extraction method developed here increased the sugar concentration in the extraction liquid with less water consumption. No comparison of energy cost between the water extraction and crushing method has yet been done for this approach. Designing a low energy consuming sugar concentrating process (possibly using solar energy) is a focus of future work.

## Conclusions

The goal of these studies was to develop an efficient sugar extraction and stalk storage method for sweet sorghum ethanol production. More than twice as much sugar was released by the water extraction method than by the more standard crushing method; and the method of sugar collection did not impact fermentation efficiency. Sugar release rate and maximum sugar released increased with increasing temperature and decreasing substrate size. A four-fold increase in sugar release rate from ground stalk at 37.8°C was achieved compared with that from the 1 cm stalk substrate at 25°C. Recycling of the sugar extraction liquid is a more efficient method with yield of 0.05 g sugar/ g fresh stalk. Stalks stored in an open field over 2 days resulted in a 20% total sugar loss under dry condition but had minimal sugar loss for storage between 2 days and 22 days. Integration of these processing methods is necessary to validate costs and efficiency but when used together, overall ethanol production efficiency should increase compared to current field practices.

## Materials and methods

### Sugar extraction procedure

The M81E cultivars of sweet sorghum cultivated and harvested by hand from the University of Arizona, Tucson Campus Agricultural Center in June 2010 and on October 15, 2010 were used for experiments. All sweet sorghum stalks and juice were stored in a −20°C freezer after harvesting. The frozen stalks were thawed at room temperature (25°C) prior to use and the rinds were removed by hand. Stalks were reduced to three sizes for this research. Size 1 stalks had a length of 1 cm. Size 2 stalks were prepared by cutting Size 1 stalks into 4 pieces along the axis direction. “Ground” stalks (Size 3) were prepared by using a 375 W home blender from Sears Roebuck and Co., Model No, 400–829301.

The extraction process was performed on a shaker at 80 rpm in an incubator for the sugar release kinetics study. Five grams of stalks was placed in 50 mL of nano-pure water in a 100 mL Erlenmeyer flask and covered with aluminum foil. Samples were taken at time points 0.5, 1, 2, 4 and 8 h. Three temperatures (25, 30, and 37.8°C) and three stalk sizes were assessed in this experiment with three replicates.

### Improved sugar extraction method

The recycled bagasse extraction experiments were conducted by adding fresh water into the bagasse recycled from a previous extraction batch. In the recycled bagasse method, three cycles of sugar extraction were performed. The bagasse was separated by a Buchner funnel. The recycled bagasse was then added to 20 ml of water to keep the same solid liquid ratio (grams of stalk/ml of water) for the next batch extraction. In the recycled liquid method, five cycles of sugar extraction were performed. The recycled water was collected after the bagasse was separated by a Buchner funnel. Five grams of fresh bagasse was then added to the recycled extraction solution to keep the same solid–liquid ratio for the next batch extraction. The batch extraction was performed at 30°C for 2 h with a shaking speed of 80 rpm. Samples were taken after each batch of extraction and analyzed for sugar (glucose, fructose, and sucrose) released.

### Sweet sorghum juice fermentation method

To test the impact on sugar content and fermentation efficiency from the filtration process, filtration using three different filters, poly2000, poly 2004 and poly 2007, provided by FLO Trend Systems Inc. (Houston, TX) was tested. The juice was fermented by Ethanol Red yeast provided by Pinal Energy, LLC (Maricopa, AZ) for 48 h in a 1.3 l BioFlo115 fermentor with inoculation size of 50 mg dry yeast/500 ml juice, agitation rate at 80 rpm at 30°C, oxygen was provided for the first two hours at a flow rate of 0.1 l per minute. Samples were removed during the fermentation process and later analyzed by HPLC for sugar and ethanol content. Methods followed those reported by Teetor [15].

### Sweet sorghum stalks storage experiment

Dry storage conditions were achieved by placing whole stalks in the open field with leaves and heads removed. The wet condition was achieved by sealing stalks in plastic bags. Ten stalks for each storage conditions were placed at University of Arizona, Tucson Campus Agricultural Center in the field for 22 days. The stalk storage test was conducted from September 29th to October 20th, 2010. The average maximum and minimum temperature was 88°F and 63°F respectively. Average temperature and relative humidity during the storage period were 75°F and 39.1% RH respectively [16]. The samples were taken by cutting off a short piece of stalk in the middle session of the whole stalk. The stalks were then pressed using a laboratory hydraulic press from Fred S. Carver, Inc. Model C, Serial No. 29000–393.

### Juice dehydration

The impact of juice dehydration (reduction in water content) was evaluated. Frozen sweet sorghum juice was thawed at room temperature and then centrifuged at 4000 rpm for 20 min to remove sediment. Water was removed by gently boiling 100 mL samples of juice on a laboratory hot plate. Reduction in juice volume from of 10, 20, 30, and 40 mL was performed. Samples were fermented one day after dehydration. The quantity of ethanol was determined by HPLC with refractive index detection after fermentation [15]. Comparisons were made to juice that had not been dehydrated. Otherwise methods used were as described above.

All comparisons in ethanol produced are shown as a relative % EtOH to the control juice which was not dehydrated. A comparison of these as prepared juice samples to control is referred to here as “variable volume” samples. When the reduction in total volume is incorporated in the analysis, the samples are termed “constant volume”.

### Sugar and ethanol concentration analyses by HPLC

The concentrations of sucrose, glucose, fructose and ethanol were measured using a Shimadzu Prominence UFLC HPLC including SIL-20A auto sampler and an RID-10A refractive index detector. A Rezex ROA-Organic Acid H + (8%) column was used for separation. The method operated at a temperature of 32°C, retention time of 30 min, and flow rate of 0.5 mL / min with 2.5 mN sulfuric acid solution used as the mobile phase [1,15]. Series concentrations of each sugar (5–50 g/L) and ethanol (5–100 g/L) standards were prepared for calibration for each day of operation.

## Competing interests

The authors declare that they have no competing interests.

## Author’s contributions

FJ participated in the design of the study and data analysis, conducted all experiments except the dehydration, and drafted the manuscript. JC performed juice dehydration experiments. MR participated in the design of the study and data analysis as well as writing the manuscript. WZ contributed on experimental design and data analysis works. KO contributed in the experimental design. All authors read and approved the final manuscript.
